# Poorly Differentiated Squamous Cell Carcinoma With Jaw Bone Osteomyelitis and Skin Perforation: A Report of a Rare Clinical Case

**DOI:** 10.7759/cureus.70738

**Published:** 2024-10-02

**Authors:** Dharini S, Karthikeyan Ramalingam, Pratibha Ramani, Rubin S John, Jayaindhraeswaran J

**Affiliations:** 1 Oral Pathology and Microbiology, Saveetha Dental College and Hospitals, Saveetha Institute of Medical and Technical Sciences, Saveetha University, Chennai, IND; 2 Oral and Maxillofacial Surgery, Saveetha Dental College and Hospitals, Saveetha Institute of Medical and Technical Sciences, Saveetha University, Chennai, IND

**Keywords:** diagnosis, head and neck, head and neck squamous cell carcinoma, mortality, oncology, oral cancer, oral squamous cell carcinoma, osteomyelitis, poorly differentiated neoplasms, quality of life

## Abstract

Oral squamous cell carcinoma is a perpetual challenge for current clinicians and oral pathologists. In this case report, we present an unusual case of oral squamous cell carcinoma involving the left buccal mucosa with extensive bone exposure and skin perforation. It showed features of ulceration, necrosis, and maggot infestation. On histological examination, the malignant epithelial cells in the dense fibrous connective tissue stroma were keratinized, highly pleomorphic, had a high nucleo-cytoplasmic ratio, and were organized in sheets, cords, and nests. Decalcified tissue sections revealed the presence of necrotic bone. Immunohistochemistry indicated diffuse PanCK (cytokeratin) positivity confirming the epithelial origin of the malignant tumor cells. A final diagnosis of poorly differentiated squamous cell carcinoma (Bryne score 13/16) with chronic osteomyelitis and skin involvement was confirmed. Palliative care with supportive therapy was recommended. Hence, this case report emphasizes how critical it is to receive an early diagnosis and treatment to stop the disease progression, prevent the host’s immune suppression, and improve the overall quality of life of the patient.

## Introduction

Over 90% of head and neck cancer cases present as head and neck squamous cell carcinoma, which is also the sixth most frequent malignancy worldwide [1,2]. The prognosis of oral squamous cell carcinoma (OSCC) is based on the patient's general health, the cancer's stage, and its location. The five-year survival rate for patients with early-stage OSCC is between 80% and 90%. In comparison, people with advanced-stage OSCC had a survival rate of between 30% and 50% [3]. The three subtypes of OSCC based on the degree of tumor cell differentiation are distinguished as, well-differentiated (WDSCC), moderately differentiated (MDSCC), and poorly differentiated (PDSCC) of which the most prevalent subtype is WDSCC.

While in PDSCC, the tumor cells are largely immature, having more mitotic figures, fewer intercellular bridges, and little to no keratinization, the tumor cells in WDSCC resemble normal squamous cells. Studies reveal that poorly differentiated squamous cell carcinoma (SCC) has a worse prognosis and a five-year overall survival rate that is 30% worse than that of the well-differentiated subtype, even though it is the uncommon subtype. Compared to patients with well-differentiated tumors, patients with poorly differentiated OSCC have significant disparities in disease features and survival, as well as a higher risk of distant metastasis and local recurrence [4]. OSCC grades determine the treatment strategy, as disease progression is a crucial aspect of malignant tumors. An accurate assessment is critical to predict overall survival in such patients [5-7].

The present case report discusses a case of poorly differentiated oral squamous cell carcinoma with a rare clinical presentation of extensive skin perforation and bone exposure concerning the left buccal mucosa.

## Case presentation

A 44-year-old man reported to the dental outpatient department with pain of a three-month duration and a large opening on his left buccal mucosa. He had noticed intermittent pain on the involved cheek for the past few weeks but did not visit any doctor. The cheek opened up suddenly a few days ago and he was referred to Saveetha Dental College for further management. The patient stated that he had a pan-chewing habit for five years. His surgical, medical, and family histories were normal. 

**Figure 1:**
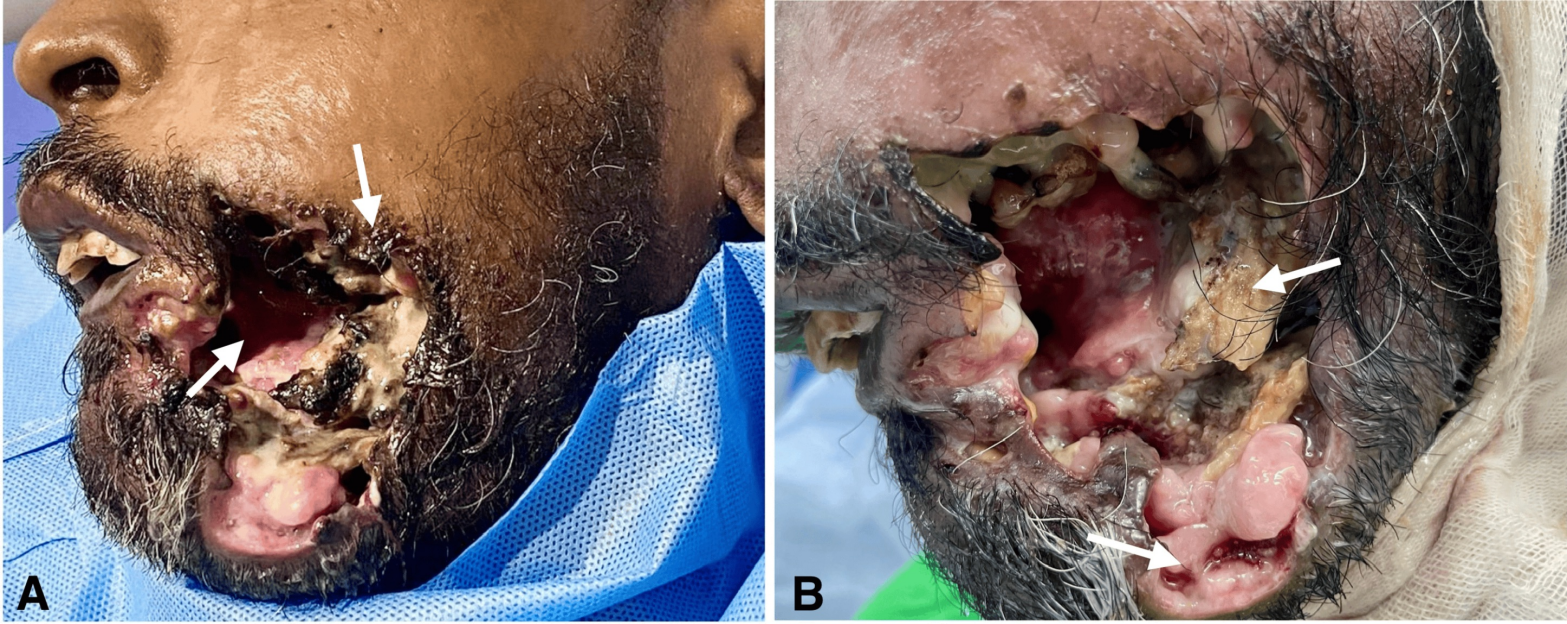
Clinical image showing (A) perforation and skin involvement and (B) soft tissue destruction and necrosed bone

On clinical examination, an approximately 7.6 x 7.2 cm large perforation was seen with irregular margins coupled with skin involvement, bone and teeth exposure, and an infestation of maggots. The face of the patient appeared necrotic with notable destruction of the adjacent soft tissue structures leaving behind raw, ulcerated, and necrotic areas. It was soft to firm and tender on palpation. It was recommended with a tentative clinical diagnosis of either SCC or NOMA (Cancrum oris) (Figure 1).

Incisional biopsy was performed under local anesthesia and tissue samples were sent to Oral Pathology Department for reporting. Two soft tissue samples and one hard tissue sample were obtained in 10% neutral buffered formalin. The specimens obtained measured 0.7 x 0.6 x 0.4 cm and were firm to hard in consistency. The color of the soft tissue was whitish brown. While the hard tissue was kept for decalcification in 5% formic acid, the soft tissue samples were processed and sectioned, and H&E-stained slides were prepared.

**Figure 2:**
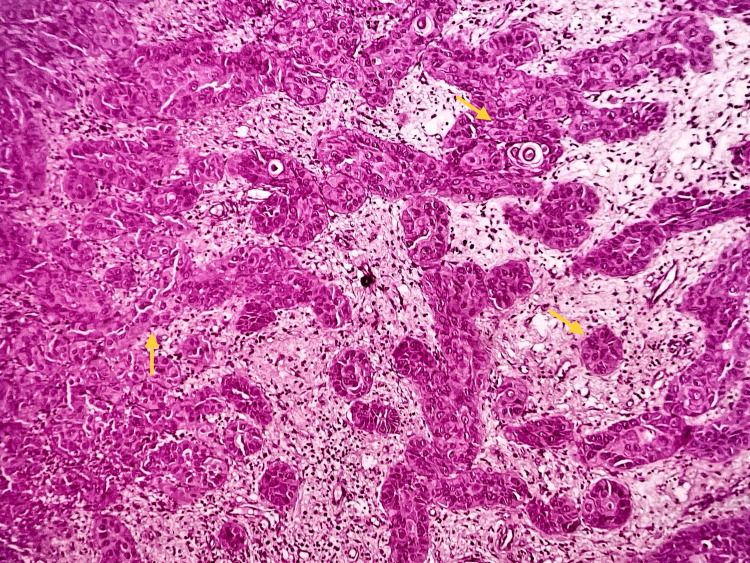
Photomicrograph of the section showing strands, cords, and islands of malignant epithelial cells (H&E, 10x)

On histopathological examination, a dense fibrous connective tissue stroma with malignant epithelial cells distributed in sheets, cords, strands, nests, and small islands of different sizes were evident. Marked nuclear and cellular pleomorphism, increased nucleo-cytoplasmic ratio, hyperchromatism and vesicular nuclei with conspicuous nucleoli, and keratinization of individual cells were all noted in the tumor cells. Significant vascularity and a moderate, diffuse mixed inflammatory cell infiltration comprising lymphocytes and neutrophils were observed. There were also several prominent areas of ulceration, necrosis, and bleeding. Necrotic bone was confirmed in decalcified H&E sections which displayed lamellar bony trabeculae with lacunae devoid of osteocytes and resorbing bone margins (Figure 2).

**Figure 3:**
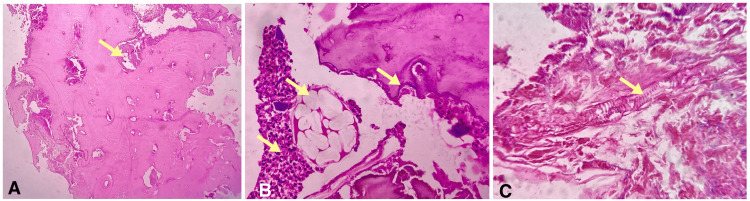
Photomicrographs of H & E stained decalcified sections showing (A) necrotic bone with empty lacunae devoid of osteocytes (H&E, 4x), (B) food particles admixed with intense acute inflammatory cells and areas of necrosis with jagged resorptive bone margins (H&E, 10x), and (C) maggot with microbial colonies and areas of hemorrhage (H&E, 10x)

There were extensive necrosis and evidence of maggots in the marrow spaces, and there was also an acute inflammatory cell infiltration composed of polymorphonuclear leukocytes (neutrophils) (Figure 3).

**Figure 4:**
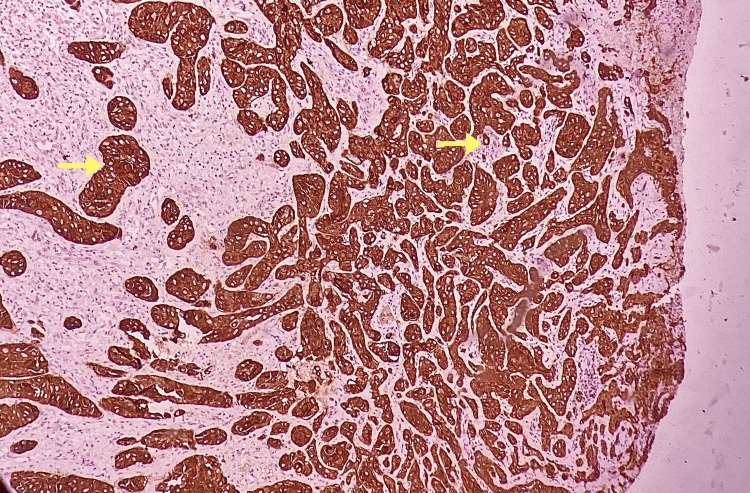
Photomicrograph showing diffuse strong positivity of pancytokeratin in all the malignant epithelial cells (IHC, 10x)

The tumor cells exhibited strong diffuse cytoplasmic positivity for PanCK (cytokeratin) as revealed by immunohistochemical examination (Figure 4).

A definitive diagnosis of poorly differentiated squamous cell carcinoma (Bryne score of 13/16) with chronic osteomyelitis was made after correlation with the clinical and histological findings. Palliative care was advised for the patient as the malignant tumor was beyond curative treatment. Stringent and continuous monitoring was recommended. The patient was lost to follow-up as he did not respond to phone calls or letters posted to his residence. 

## Discussion

The most prevalent histological subtype reported was well-differentiated squamous cell carcinoma followed by moderately differentiated SCC and poorly differentiated SCC was noted only among 2.94% of patients [6]. Studies on poorly differentiated oral and oropharyngeal cancers have shown that the location of the tumor, disease stage, and lymph node metastases are important survival variables [7]. Because many individuals arrive with advanced-stage disease, these findings highlight the need for early identification and preventative treatments.

Genetic mutations and epigenetic changes in signaling pathways accumulate to initiate OSCC occurrence which begins as dysplasia, develops into carcinoma in situ, and ultimately becomes malignant. The patient under discussion in the case report disclosed a five-year history of pan chewing habit linked to the increased risk of OSCC occurrence. Oral submucous fibrosis, lichen planus, syphilis, smoking, alcohol consumption, betel nut chewing, poor dental hygiene, and malnourishment are all major contributors to the development of OSCC particularly on the Asian subcontinent. Previous research suggests that a major factor in the increased incidence of tongue and oral malignancies in the Indian subcontinent is the combination of malnourishment and betel nut eating consistent with the current case presented [8].

Long-term pan usage, which includes flavonoids, the alkaloid arecoline, and areca nut, can cause submucous fibrosis and leukoplakia. Reactive oxygen species produced by betel quid cause oxidative stress in the tissues around them, further damaging DNA [9]. Malnutrition and arecoline together further impair immunity, which aids in the development of cancer. Furthermore, smoking releases carcinogenic alkaloids, which are a significant contributing factor to the development of OSCC, especially when combined with slaked lime [10].

In this case, the patient's unawareness, uncooperativeness, and ignorant behavior resulted in delayed diagnosis, poor prognosis, and lack of treatment. The disease's painless nature, the challenge of examining lesions in the posterior third of the tongue, low socioeconomic status, poor oral hygiene, ignorance of dental issues, and professional delays brought on by a lack of appropriately trained dental professionals in remote areas are some additional contributing factors that have been highlighted in prior research [11]. Signs of skin involvement, lymph node metastases, bone invasion, and local aggressiveness are common in late-stage presentations [12]. Similarly, the patient in this report had severe skin involvement, exposed bone, and poor oral hygiene. Histopathology revealed necrosis and a maggot infestation which is a rare presentation. 

Compared to a PDSCC without skin involvement, skin invasion in OSCC is associated with a poorer prognosis. This is classified as a type of dermal metastasis. Research has indicated that individuals with cutaneous or mandibular cortex invasion and locally progressed OSCC have worse overall survival (52% with invasion vs. 65% without) and disease-specific survival (69% with invasion vs. 79% without) over five years [13]. According to reports, quality of life declines significantly and rapidly when face skin involvement is apparent at diagnosis [14]. Therefore, it is crucial for determining prognosis and directing treatment choices as skin invasion has been reported to have an independent impact on survival in advanced OSCC.

The mean quality of life index consistently indicated a decline in the postoperative phase compared to preoperative levels, underscoring the lasting impact of oral cancer and its treatment on patients [15]. Given the numerous risk factors associated with OSCC, previous research has highlighted the importance of quality of life for these patients [16]. The recent development of a quality-of-life prediction model for oral cancer patients offers a valuable tool for improving planning and treatment strategies. This model supports a personalized approach to care, enhances patient empowerment, and optimizes resource allocation for high-quality, patient-centered treatment [17]. Additionally, systematic reviews and audits of patient records can pinpoint areas for treatment improvement, identify training needs, and address potential risks [18,19]. The case described in this report is aggressive, emphasizing how urgently a professional intervention is required. Hence, early identification is essential since it substantially impacts immunological suppression, the course of the disease, and the patients' overall quality of life. 

## Conclusions

The large extent of the lesion and its distinctive presentation such as necrosis, bone exposure, and the presence of maggots, and legumes make this case particularly unique. Immunohistochemical studies corroborated the diagnosis of epithelial origin. This case's forceful presentation underscores how crucial it is for the patient to seek early diagnosis and for the medical professionals to intervene as soon as possible. Early diagnosis and intervention are crucial because postponements can worsen the course of the condition, weaken the immune system, and negatively affect the patient's quality of life as indicated in this case.
